# New Interactors of the Truncated EBNA-LP Protein Identified by Mass Spectrometry in P3HR1 Burkitt’s Lymphoma Cells

**DOI:** 10.3390/cancers10010012

**Published:** 2018-01-05

**Authors:** Sonia Chelouah, Emilie Cochet, Sophie Couvé, Sandy Balkaran, Aude Robert, Evelyne May, Vasily Ogryzko, Joëlle Wiels

**Affiliations:** 1 UMR 8126 CNRS, Univ. Paris-Sud, Université Paris-Saclay, Institut Gustave Roussy, F-94805 Villejuif, France; chelouah_sonia@yahoo.fr (S.C.); sandy_bal22@hotmail.com (S.B.); aude.robert@gustaveroussy.fr (A.R.); may.evelyne@wanadoo.fr (E.M.); 2 Proteomic Platform, Institut Gustave Roussy, Villejuif F-94805, France; emilie.cochet@servier.com; 3 Laboratoire de Génétique Oncologique, Ecole Pratique des Hautes Etudes, F-75014 Paris, France; Sophie.COUVE@gustaveroussy.fr; 4 UMR 1186 Inserm, Univ. Paris-Sud, EPHE, Université Paris-Saclay, Institut Gustave Roussy, F-94805 Villejuif, France

**Keywords:** EBV, Wp-restricted latency, mass spectrometry, EBNA-LP, PP2A, SSRP1, TFEC

## Abstract

The Epstein-Barr virus nuclear antigen leader protein (EBNA-LP) acts as a co-activator of EBNA-2, a transcriptional activator essential for Epstein-Barr virus (EBV)-induced B-cell transformation. Burkitt’s lymphoma (BL) cells harboring a mutant EBV strain that lacks both the EBNA-2 gene and 3′ exons of EBNA-LP express Y1Y2-truncated isoforms of EBNA-LP (tEBNA-LP) and better resist apoptosis than if infected with the wild-type virus. In such BL cells, tEBNA-LP interacts with the protein phosphatase 2A (PP2A) catalytic subunit (PP2A C), and this interaction likely plays a role in resistance to apoptosis. Here, 28 cellular and four viral proteins have been identified by mass spectrometry as further possible interactors of tEBNA-LP. Three interactions were confirmed by immunoprecipitation and Western blotting, namely with the A structural subunit of PP2A (PP2A A), the structure-specific recognition protein 1 (SSRP1, a component of the facilitate chromatin transcription (FACT) complex), and a new form of the transcription factor EC (TFEC). Thus, tEBNA-LP appears to be involved not only in cell resistance to apoptosis through its interaction with two PP2A subunits, but also in other processes where its ability to co-activate transcriptional regulators could be important.

## 1. Introduction

Epstein-Barr virus (EBV) belongs to the human gamma herpes virus family that was initially isolated from a Burkitt's lymphoma (BL) cell line [1]. Later, EBV was shown to be associated with other malignancies such as Hodgkin lymphoma, post-transplant lymphoproliferative disorders (PTLD), and nasopharyngeal carcinoma (NPC). The infection of B lymphocytes in vitro by EBV generates immortalized lymphoblastoid cell lines (LCLs). In latently-infected cells, EBV produces nine viral proteins, including six nuclear antigens (EBNA-1, -2, -3A, -3B, -3C and -LP) and three membrane proteins (LMP-1, 2A, and 2B), as well as two small non-coding RNAs (EBER1 and EBER2). EBV has three different latency programs, characterized by the expression of distinct combinations of proteins. Most BL cells show the latency I phenotype, in which EBNA-1 is expressed alone from the viral Q promoter (Qp) [2,3]. However, Kelly et al. showed that approximately 15% of BL tumors display an atypical latency program characterized by the exclusive usage of the W promoter (and therefore called “Wp-restricted” latency). These tumors carry a variant genome lacking both the EBNA-2 gene and the unique Y1Y2 exons of EBNA-LP. They express EBNA-1, EBNA-3A, 3B, 3C and a truncated form of EBNA-LP (tEBNA-LP) [4]. This EBV variant was first described in two long-established BL lines: P3HR1 and Daudi [5].

Our laboratory and others have shown that these Wp-restricted BL cells are more resistant to apoptosis than those infected by wild-type EBV. Various viral proteins seem to have anti-apoptotic properties. Kelly et al. first suggested that the EBNA-3 proteins are involved in this apoptotic resistance because these viral antigens are consistently found in Wp-restricted BL cells [6]. However, they later reported that this resistance is mediated by the Wp-driven expression of the viral Bcl2 homolog BHRF1: a protein usually associated with the virus lytic cycle [7]. Another group also showed that BHRF1 functions as a survival factor in the Wp-restricted cell line P3HR1 [8]. On the other hand, we found that the tEBNA-LP protein is also involved in apoptotic resistance. Indeed, we showed that various Wp-restricted cell lines that are resistant to verotoxin-induced apoptosis produce low molecular weight isoforms of tEBNA-LP, which can interfere with the activation of the protein phosphatase 2A (PP2A) involved in this apoptotic pathway [9,10].

Full-length EBNA-LP, also known as EBNA-5 [11,12], is encoded by a variable number of two repeated exons (W1-W2) located in the viral internal repeated region 1 (IR1), and by two unique 3′ exons (Y1-Y2) which are deleted in tEBNA-LP [13,14]. The EBNA-LP gene is transcribed from Wp during the early stages of infection, and from the C promoter (Cp) (upstream from Wp) during the later stages of infection or in LCLs [15,16,17]. In BL cells carrying the variant EBV genome, tEBNA-LP is transcribed exclusively from Wp [4].

EBNA-LP is involved in the immortalization of B cells mostly because it co-activates the transcriptional activator EBNA-2. Two studies have shown that this function maps to the W1W2 repeated domains of EBNA-LP. Indeed, in co-transfection experiments, tEBNA-LP and full-length EBNA-LP stimulate the transcriptional activity of EBNA-2 to the same extent. These results suggested that an EBV strain lacking only the Y1-Y2 exons of EBNA-LP should be able to immortalize B lymphocytes [18,19]. However, further studies demonstrated that such recombinant viruses are either less efficient or markedly impaired in their ability to transform B lymphocytes [20,21].

EBNA-LP also interacts directly with several cellular proteins, including tumor suppressors and proteins involved in apoptosis or cell cycle regulation (for a review see [22]). Some of these interactions are mediated by the repeated W1W2 N-terminus of the protein and therefore may be preserved in tEBNA-LP. This is notably the case for HAX-1 (a negative regulator of apoptosis [23]) and DNA-PKcs (one component of a complex involved in DNA repair [24]). EBNA-LP also interacts with the heat shock protein HSP72 but the domain of EBNA-LP involved in this interaction is still unclear. This is because Mannick et al. showed that HSP72 associates with the EBNA-LP W1W2 repeated domains [25], whereas two other groups reported that it interacts with the Y1Y2 C-terminal domain [26,27]. All these interactions were detected in cells transfected with expression vectors encoding either the full-length or the Y1Y2-deleted form of EBNA-LP.

In this study, we aimed to identify interactors of the endogenous form of tEBNA-LP in its physiological environment. We immunoprecipitated tEBNA-LP from P3HR1 BL cells and analyzed its partners by mass spectrometry. With this method, we confirm that tEBNA-LP interacts with PP2A, and show that three of the previously identified full length EBNA-LP-binding partners [28] also interact with endogenous tEBNA-LP. We uncover 32 new putative interactors, including four viral proteins. Functional annotation of these interactors show that they are involved in various cellular and viral functions, including cell death, cellular growth and proliferation, gene expression, and EBV DNA replication. Finally, we validate the interaction of tEBNA-LP with three of these proteins by immunoprecipitation followed by Western blotting.

## 2. Results and Discussion

### 2.1. Production of Recombinant EBNA-LP and Identification of Its Specific Peptides by Mass Spectrometry

**Figure 1:**
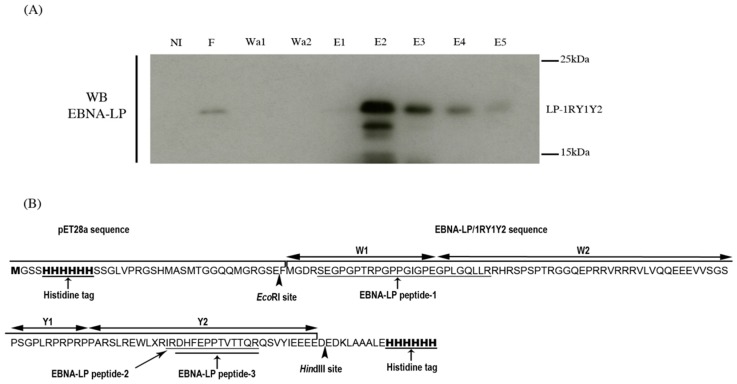
Production of recombinant Epstein-Barr nuclear antigen-leader protein (EBNA-LP) and identification of its specific peptides by mass spectrometry. (**A**) Various fractions obtained during affinity chromatography of isopropyl β-D-1thiogalactopyranoside (IPTG)-induced or non-induced bacterial lysates were analyzed by Western blotting with 4D3 anti-EBNA-LP monoclonal antibody (mAb). NI: non-induced bacterial lysate; F: flow-through; Wa: washing; and E: eluted fractions of induced bacterial lysates. (**B**) EBNA-LP specific peptides identified by mass spectrometry and their location on the sequence of the recombinant protein 6× His-EBNA-LP-1RY1Y2.

To identify peptides of EBNA-LP that can be used in mass spectrometry analysis, we first produced a 6× His-tagged-recombinant protein containing one W1W2 repeat and the unique Y1Y2 C-terminal domain (LP-1RY1Y2). The sequence was cloned in the pET28a vector and expressed in the *Escherichia coli* Rosetta (DE3) strain. The 6× His-tagged LP-1RY1Y2 was purified by affinity chromatography using Ni-TED (Tris carboxymethyl ethylene diamine) columns. Washed and eluted fractions were separated by electrophoresis on preparative sodium dodecyl sulphate-polyacrylamide gel electrophoresis (SDS-PAGE) stained with Coomassie blue. A Western blot probed with an anti-EBNA-LP antibody was performed in parallel on an analytical gel (Figure 1A). This enabled us to localize the LP-1RY1Y2 Coomassie-blue stained band on the preparative gel. This band was cut, digested with trypsin and analyzed by LC-MS/MS.

Three EBNA-LP specific peptides were detected. Peptide-1, (SEGPGPTRPGPPGIGPEGPLGQLLR) is located at the junction between the W1 and W2 domains of EBNA-LP whereas the two others (IRDHFEPPTVTTQR and DHFEPPTVTTQR) are located in the Y2 domain (Figure 1B). Therefore, only peptide-1 can be used to validate the presence of tEBNA-LP in the immunoprecipitates analyzed by LC-MS/MS analysis.

### 2.2. Identification of Truncated EBNA-LP Partners by Mass Spectrometry

To characterize tEBNA-LP partners, we immunoprecipitated protein extracts of P3HR1 cells with the 4D3 anti-EBNA-LP mAb or with an irrelevant mouse IgG as a negative control. The immunoprecipitates were then separated by SDS-PAGE electrophoresis, the gels were stained with Coomassie blue and each lane was cut into 24 pieces. Proteins contained in these pieces were "in gel-digested" by trypsin and the peptides were analyzed by LC-MS/MS. Five independent experiments were performed and analyzed. Positive hits were defined as proteins which were found in the anti-EBNA-LP immunoprecipitates and not in the control IgG immunoprecipitates, in at least two independent experiments. Table 1 lists the 32 potential interactors of tEBNA-LP identified, which include 28 cellular and four viral proteins. Among the cellular proteins, we found the structural subunit A of PP2A which confirms our previous finding that PP2A interacts with tEBNA-LP [9].

Most (21/28) of the cellular partners of tEBNA-LP are intracellular proteins, including cytoplasmic proteins like FAP1 and CLASP1, nuclear proteins like TFEC, SSRP1 and SF3B14 or, similar to EBNA-LP itself, proteins that are present in both compartments (e.g., iNOS and PP2A). Indeed, EBNA-LP contains a bipartite nuclear localization signal located in the W2 domain [29], but isoforms of EBNA-LP smaller than 42 kDa can shuttle between the nucleus and the cytoplasm by simple diffusion [30]. We also showed previously that low molecular weight forms of tEBNA-LP expressed in cells resistant to apoptosis are predominantly cytoplasmic but can also be detected in the nucleus [9]. Therefore, tEBNA-LP may interact with proteins localized in the cytoplasm or the nucleus. The rest (7/28) of the potential partners of tEBNA-LP, like fibronectin, laminin or fibrinogen, are extracellular proteins. This result is somewhat surprising since tEBNA-LP lacks a signal peptide and cannot be secreted by the conventional ER to Golgi pathway of protein secretion. However, it remains possible that tEBNA-LP, like other small proteins, is secreted by an unconventional mechanism [31]. Yet, to our knowledge, no data has been reported on this topic. Therefore, further studies are needed to confirm interactions between tEBNA-LP and extracellular proteins.

Among the 147 putative interactors of full-length EBNA-LP identified by Forsman et al. [28] through a modified tandem affinity purification (TAP) method followed by LS-MS/MS analysis, we found only three (L-lactate dehydrogenase A and B chains and Heat shock protein HSP 90) that potentially interact with tEBNA-LP. This suggests that the 25 other cellular proteins listed in Table 1 specifically interact with tEBNA-LP, especially given that many of the binding partners identified by Forsman et al., including Hsp70, Hsc70, tubulin α and β, HAX-1 and HA95, were previously reported to interact with EBNA-LP.

We also found that tEBNA-LP interacts with the following viral proteins in P3HR1 cells: BBRF1, which is involved in viral genome packaging; BORF2, the large subunit of the ribonucleoside-diphosphate reductase holoenzyme which is necessary for viral DNA synthesis; the serine-threonine kinase BGLF4, which phosphorylates numerous cellular and viral (including EBNA-LP) proteins and is required for efficient viral DNA replication [32]; and EBNA-3B, a nuclear protein that regulates cellular genes, alone or in cooperation with the other EBNA-3 (A and C) [33,34]. Given that the expression of the EBNA-3 proteins may be also involved in the resistance of Wp-restricted BL cells to apoptosis [6], it would be interesting to confirm these interactions.

### 2.3. Functional Annotation of tEBNA-LP Partners

To investigate the functional importance of the interactions between tEBNA-LP and its putative partners, we annotated these proteins by the IPA software of Ingenuity systems (http://www.ingenuity.com). These interactors are involved in many cellular functions (listed in Table 2), including cell death and transcriptional regulation, two functions already attributed to tEBNA-LP [22]. The partners of tEBNA-LP involved in cell death are PP2A, PTPN13/FAP1, SSRP1, iNOS, FN1, LAMA4, HSPE1, EZR, SF3B14, A2M, LDHA, and MLL. The partners of tEBNA-LP involved in transcriptional regulation are TFEC, SSRP1, FN1, A2M, and iNOS. We also identified other interactors involved in functions not previously attributed to tEBNA-LP, including cellular growth and proliferation, cellular development, cell signaling, cellular movement, cell-to-cell signaling and interaction, post-translational modifications, cell morphology, and amino acid metabolism.

Finally, we classified the partners of tEBNA-LP into the disease categories listed in IPA software, which showed that they are involved in genetic disorders, cancer, neurological disease, muscular disorders, and hematological diseases (Supplementary Materials, Table S1).

### 2.4. Validation of Novel Protein Interactions Identified by Mass Spectrometry

We used immunoprecipitation followed by Western blotting to validate the interactions of tEBNA-LP with three proteins that are involved in cell death and transcriptional regulation: PP2A, SSRP1, and TFEC.

#### 2.4.1. Truncated EBNA-LP interacts with the structural subunit of PP2A

**Figure 2:**
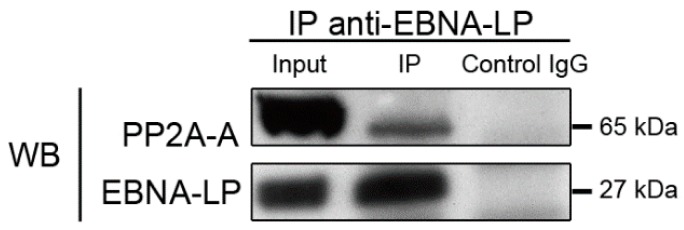
Structural subunit of protein phosphatase 2A (PP2A) co-precipitates with truncated EBNA-LP. P3HR1 cell lysates were immunoprecipitated with 4D3 anti-EBNA-LP mAb (IP) or control mouse IgG (IgG control) and the immunoprecipitates were analyzed by Western blotting with 4D3 mAb or an anti-PP2A A pAb. As a control for protein levels before IP, a portion of cell lysates (input) corresponding to 10% of the input for IP was also included in the Western blot. All results are representative of three independent experiments.

We previously showed that the catalytic C subunit of PP2A co-precipitates with tEBNA-LP, and that this interaction may lead to the inactivation of the phosphatase [9]. Here we show, through mass spectrometry analysis of proteins co-precipitating with tEBNA-LP, that the A structural subunit of PP2A (PP2A A) also interacts with tEBNA-LP (see Table 1 and Figure S1). To confirm this interaction, proteins extracted from P3HR1 cell were immunoprecipitated with 4D3 anti-EBNA-LP mAb and the immunoprecipitates were analyzed by Western blotting with anti-PP2A A pAb and anti-EBNA-LP mAb (Figure 2). The structural subunit of PP2A was detected in the 4D3 immunoprecipitates, thus confirming that endogenous tEBNA-LP interacts with the serine/threonine phosphatase PP2A in P3HR1 cells.

This phosphatase, which consists of a heterodimeric (A/C) core enzyme associated with a variable regulatory subunit (B), is involved in many essential cellular functions such as cell-cycle regulation, cell-growth control, development, signal transduction, cytoskeletal dynamics, and cell mobility [35]. PP2A is also involved in cell death: it co-localizes with Bcl-2 at the mitochondrial membrane to ensure its dephosphorylation in response to many apoptotic stimuli [36]; it interacts with and dephosphorylates Bax, thereby enhancing its pro-apoptotic function [37]. We also showed previously that treatment of BL cell lines with verotoxin-1 (VT-1) activates PP2A which leads to the re-localization of Bax to the mitochondrial membrane [10]. Given its role in maintaining cell homeostasis, the phosphatase activity of PP2A in cancer cells is often inhibited by various proteins. Among these proteins, CIP2A (Cancerous Inhibitor of PP2A) is overexpressed in a huge variety of tumors [38], whereas SET/I2PP2A (inhibitor 2 of PP2A) is only found to be overexpressed in hematopoietic malignancies. Overexpression of both proteins is associated with poor prognosis [20]. Therefore, pharmacological compounds that restore PP2A tumor suppressor activity or inhibit its interactors are now considered to be promising therapeutic molecules [39]. PP2A is also targeted by various viral proteins encoded by oncogenic viruses such as the adenovirus E4orf4 protein, the polyomavirus small and middle T antigens, and the SV40 small t antigen [40,41,42,43]. Our results add tEBNA-LP to the list of viral proteins interacting with PP2A. SV40 small t antigen interacts with the A subunit of PP2A at a site which overlaps with the binding site of the regulatory B subunit and thereby alters PP2A substrate specificity and phosphatase activity. Here, we show that tEBNA-LP stably interacts with the A subunit of PP2A, while we had previously shown that the catalytic C subunit of PP2A coprecipitates with tEBNA-LP [9]. Together these results suggest that the interaction of tEBNA-LP with the core complex of PP2A could destabilize the holoenzyme and thus inactivate PP2A.

#### 2.4.2. Truncated EBNA-LP Interacts with TFEC

**Figure 3:**
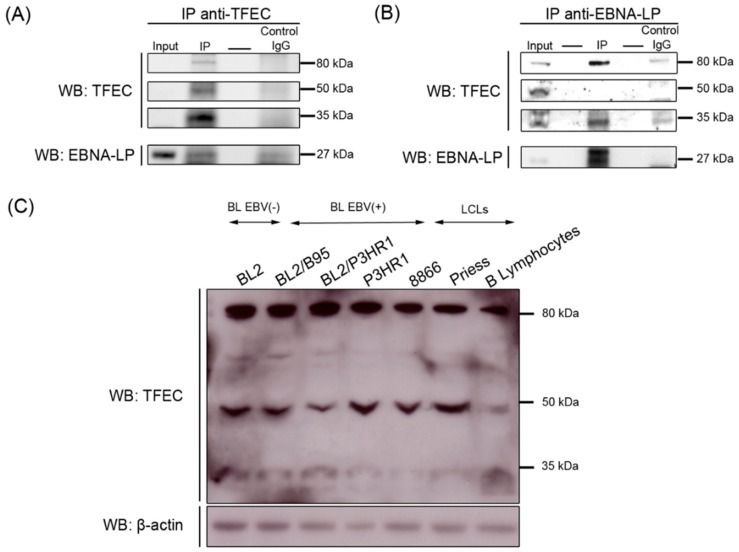
Truncated EBNA-LP interacts with different isoforms of transcription factor EC (TFEC). (**A**) P3HR1 cell lysates were immunoprecipitated with L-15 anti-TFEC pAb (IP) or control goat IgG (IgG control) and the immunoprecipitates were analyzed by Western blotting with L-15 pAb or 4D3 anti-EBNA-LP mAb. (**B**) P3HR1 cell lysates were immunoprecipitated with 4D3 anti-EBNA-LP mAb (IP) or control mouse IgG (IgG control) and the immunoprecipitates were analyzed by Western blotting with 4D3 mAb or an anti-TFEC pAb. As a control for protein levels before IP, a portion of cell lysates (input) corresponding to 10% of the input for IP was also included in the Western blot. (**C**) Cell lysates prepared from different B cell lines or normal B lymphocytes were submitted to Western blot analysis for detection of the various isoforms of TFEC. All results are representative of three independent experiments.

Transcription factor EC (TFEC) belongs to the microphtalmia family (MITF) of basic helix-loop-helix leucine zipper transcription factors [44,45]. The analysis by mass spectrometry of proteins co-immunoprecipitated with the anti-EBNA-LP mAb identified a TFEC-specific peptide in three bands migrating around 35, 50 and 80 kDa on the polyacrylamide gels. Proteins migrating around 35 kDa corresponds to the theoretical molecular weight of the two main human TFEC isoforms produced by alternative splicing [45,46], whereas the two other bands of higher molecular weight may correspond to post-translationally modified forms of the protein. To validate the interaction between tEBNA-LP and these three putative forms of TFEC, proteins extracted from P3HR1 cells were first immunoprecipitated with L-15 anti-TFEC pAb. The resultant immunoprecipitates were then analyzed by Western blotting with anti-TFEC pAb and anti-EBNA-LP mAb. All three forms (35, 50 and 80 kDa) of TFEC were present in P3HR1 cells and a small amount of tEBNA-LP coprecipitated with TFEC (Figure 3A). Next, we immunoprecipitated P3HR1 protein extracts with the 4D3 anti-EBNA-LP mAb and analyzed the immune complexes by Western blotting with the anti-TFEC and anti-EBNA-LP antibodies. The 80 and 35 kDa but not the 50 kDa form of TFEC coprecipitated with tEBNA-LP (Figure 3B).

To determine whether the three forms of TFEC are expressed in other B cells, we extracted proteins from normal B lymphocytes, four BL cell lines (BL2, BL2/B95, BL2/P3HR1, and P3HR1), and two LCLs (RPMI8866 and Priess) and performed Western blotting with L-15 anti-TFEC pAb (Figure 3C). Although their expression levels varied between cell samples, we detected the three forms of TFEC in all types of cells, regardless of their tumor and EBV status. Altogether, these results indicate that, in addition to the previously described 35 kDa form of TFEC, both normal and tumoral B cells contain two higher molecular weight forms of this protein, and that the 35 and 80 kDa forms interact with the tEBNA-LP protein in P3HR1 cell line.

The other members of the MITF family (MITF, TFE3, and TFEB) are sumoylated in vivo and the sumoylation of MITF regulates its transcriptional activity [47,48]. Recently, Bertolotto et al. showed that individuals with a high risk of developing melanoma, renal carcinoma or both, have a germline mutation of MITF that severely impairs its sumoylation, with a frequency significantly higher than the controls [49]. Together, these data suggest that the transcriptional regulation of MITF family members by SUMO modifications are involved in oncogenesis. So far, the sumoylation of TFEC has not been reported but computational analysis, using SUMOsp 2.0 software [50], of the TFEC amino-acid sequence identified three consensus sumoylation sites (ΨK*X*E). Interestingly, one of these sites is at a similar position relative to the basic-helix-loop-helix domain as the sumoylation sites in MITF and TFE3. It would, therefore, be interesting to test whether the higher molecular weight forms of TFEC are sumoylated.

#### 2.4.3. Truncated EBNA-LP Interacts with SSRP1

**Figure 4:**
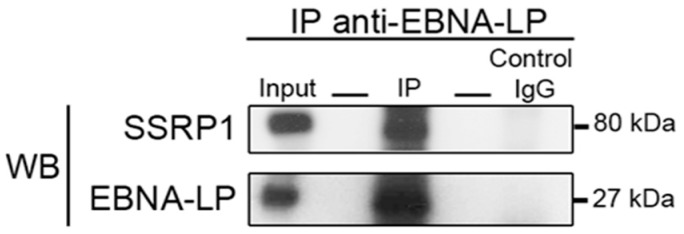
SSRP1 co-precipitates with truncated EBNA-LP. P3HR1 cell lysates were immunoprecipitated with 4D3 anti-EBNA-LP mAb (IP) or control mouse IgG (IgG control) and the immunoprecipitates were analyzed by Western blotting with 4D3 mAb or an anti-SSRP1 mAb. As a control for protein levels before IP, a portion of cell lysates (input) corresponding to 10% of the input for IP was also included in the Western blot. All results are representative of three independent experiments.

SSRP1 (Structure-Specific Recognition Protein 1) is a component of the transcription and replication FACT (Facilitator of Chromatin Transcription) complex [51]. It contains a single DNA-binding high mobility group (HMG) domain that specifically binds cisplatin-modified DNA [52,53]. To confirm the interaction between tEBNA-LP and SSRP1, we immunoprecipitated total lysates of P3HR1 cells with 4D3 anti-EBNA-LP mAb and performed Western blotting to probe for EBNA-LP and SSRP1. SSRP1 co-precipitated with tEBNA-LP (Figure 4), thus confirming the interaction between these two proteins.

Previous studies have revealed an SSRP1 association with various viral proteins involved in DNA replication or transcription. For example, SSRP1 has been shown to interact with latency-associated nuclear antigen (LANA) of the Kaposi’s sarcoma-associated herpesvirus (KSHV), and thus to directly contribute to LANA-dependent DNA replication of the KSHV latent origin [54]. In accordance with our results for tEBNA-LP, it was found that SSRP1, but not Spt16 (the other component in the FACT complex), associates with LANA. These observations are consistent with a previously demonstrated Spt16-independent role of SSRP1, making it possible that additional SSRP1-containing complexes exist with other partners [55]. On the other hand, SSRP1 has been recently shown to interact with ICP22: one among the four immediate early proteins of Herpes virus simplex 1 (HSV-1) involved in the regulation of the viral transcription carried out by cellular RNA polymerase II [56]. It is generally considered that the principal function of EBNA-LP is to co-activate a subset of EBNA-2-regulated viral latency genes [57], but EBNA-LP could well exert its co-activation function as part of other protein complexes, especially in the absence of EBNA-2. Our data support this hypothesis by showing that tEBNA-LP interacts with SSRP1, one of whose roles is to increase the efficiency of transcription while preserving chromatin structure.

## 3. Materials and Methods

### 3.1. Cell Lines

BL cell lines were originally established from endemic or sporadic cases of BL. P3HR1 BL cells which contain a variant EBV strain were provided by G. Klein (Stockholm). BL2/B95 and BL2/P3HR1 were generated by stable infection of the original EBV-negative BL2 cells with the B95.8 and P3HR1 virus strains, respectively. These cells were kindly provided by the International Agency for Research on Cancer (IARC, Lyon, France). The LCLs, RPMI8866 and Priess, obtained by the in vitro immortalization of normal B lymphocytes, were provided by IARC and Julia G. Bodmer (Imperial Cancer Research Fund, London, UK), respectively. Normal B lymphocytes were provided by Yegor Vassetzky (our laboratory). These cell lines were cultured in RPMI 1640 medium (PAA) containing 2 mM L-glutamine, 1 mM sodium pyruvate, 20 mM glucose, 100 U/mL penicillin and 100 mg/mL streptomycin and supplemented with 10% heat-inactivated fetal calf serum.

### 3.2. Antibodies

Anti-EBNA-LP (4D3) monoclonal antibody (mAb) was kindly provided by Yasushi Kawaguchi (Division of molecular virology, University of Tokyo, Tokyo, Japan) [58]. Anti-PP2A A (07-250) rabbit polyclonal Ab was purchased from Millipore (Guyancourt, France), anti-SSRP1 (clone 3E4) mouse mAb from Biolegend (San Diego, CA, USA), anti-TFEC (L-15) goat polyclonal Ab from Santa Cruz Biotechnology (Heidelberg, Germany) and anti-β actin (AC-74) mAb from Sigma-Aldrich (Saint-Quentin Fallavier, France). Horseradish peroxidase (HRP)-conjugated rabbit anti-mouse IgG, HRP-conjugated donkey anti-rabbit IgG and HRP-conjugated donkey anti-goat IgG used for Western blotting were purchased from Zymed (Villebon-sur-Yvette, France), GE Healthcare (Saint-Quentin Fallavier, France) and Santa Cruz Biotechnology, respectively.

### 3.3. Western Blot Analysis

Aliquots of 1 × 10^6^ cells were pelleted and lysed in ice-cold radio-immunoprecipitation assay (RIPA) lysis buffer (150 mM NaCl, 50 mM Tris pH 7.4, 5 mM EDTA, 1% NP40, 0.5% NaDoc, 0.1% SDS, Complete 1×). Sample loading buffer was added and the mixture was boiled for 5 min. Equal amounts of protein were then separated by electrophoresis on appropriate Bis-Tris precast gels (Invitrogen, Villebon-sur-Yvette, France) and transferred to PVDF membranes (Millipore) by electroblotting. Blots were blocked overnight at 4 °C in 3% nonfat milk powder and 2% glycine in PBS, and then incubated for 1 h at room temperature or overnight at 4 °C with primary antibodies. After repeated washing, blots were incubated with appropriate HRP-conjugated secondary antibodies. Antibody complexes were detected with a chemiluminescent HRP substrate (Millipore).

### 3.4. Immunoprecipitation

Cells (5 × 10^6^) were lysed in 1 mL of 1% NP40 buffer (20 mM Tris HCl pH 7.4, 130 mM NaCl, 2 mM EDTA, 10% glycerol, 1% NP40, Complete 1×). The lysates were mixed with 2 µg of specific antibody or IgG control antibody, incubated overnight at 4 °C under end-over-end rotation and then for 4 h at 4 °C with 20 µL of agarose-conjugated protein G or A (Sigma-Aldrich). Immune complexes were washed twice with ice-cold 1% NP40 buffer, twice with PBS and resuspended in loading buffer. The samples were boiled for 5 min and the proteins were separated on Bis-Tris precast gels and analyzed by Western blotting or used for mass spectrometry analysis.

### 3.5. Production and Purification of 6xHis-EBNA-LP-1RY1Y2

The sequence coding for EBNA-LP-1RY1Y2 (one W1W2 repeat and the unique Y1Y2 C-terminal domain) contained in the pSG5-1RY1Y2 (kindly provided by Andrew Bell, Birmingham, UK), was amplified by PCR with the following oligonucleotides: 5′-AAAAGAATTCATGGGAGACCGAAGTGAAGG-3′, which is complementary to sequences in W1 and contains an *Eco*RI restriction site (underlined) and an in-frame ATG initiation codon, and 5′-AAAAAAGCTTGTCTTCATCCTCTTCTTC-3′, which is complementary to sequences in Y2 and contains a *Hin*dIIII restriction site (underlined). The PCR product was then cloned into a pET28a plasmid digested with *Eco*RI/*Hin*dIIII and sequenced. The resulting pET28a-1RY1Y2 plasmid was used to transform the *E. coli* Rosetta (DE3) strain kindly provided by Murat Saparbaev (UMR 8200 CNRS, Villejuif, France). Production of recombinant 6× His-EBNA-LP-1RY1Y2 was induced by treating bacteria with 0.5 mM IPTG (isopropyl β-D-1thiogalactopyranoside) during 2 hours at 30 °C. After lysis of bacteria, 6× His-EBNA-LP-1RY1Y2 was then purified on Protino Ni-TED/Ni-IDA columns as recommended by the manufacturer (Macherey-Nagel, Hoerdt, France).

### 3.6. Identification of EBNA-LP Specific Peptides by Mass Spectrometry

After purification on columns, the eluted protein fractions were separated on two electrophoresis gels: one gel was transferred to a membrane and Western blotting was carried out with an anti-EBNA-LP antibody, and the other gel was stained with Coomassie blue (Invitrogen) for 1 h at room temperature and then washed with sterile water overnight at room temperature. The recombinant protein was in gel-digested by trypsin and the peptides generated were analyzed by LC-MS/MS, as described in the following section.

### 3.7. Identification of tEBNA-LP Partners by Mass Spectrometry

The endogenous truncated EBNA-LP expressed by P3HR1 cells was immunoprecipitated (IP) with anti-EBNA-LP antibody (4D3) using the protocol described above. After elution, the samples were analyzed by gel-LC-MS/MS. First, the proteins were separated on SDS-PAGE gel (Invitrogen). The gel was stained with Coomassie blue for 1 h at room temperature and then washed with sterile water overnight at room temperature. The three tracks of the gel (Input, IP, and IRR) were cut into several continuous pieces. The proteins contained in each piece were in gel-digested by trypsin as described previously [59]. Briefly, the gel slices were dehydrated with 300 µL of 50% acetonitrile followed by 300 µL of 100% acetonitrile, then re-hydrated with 300 µL of 50 mM ammonium bicarbonate. A final dehydration step was performed with 2 washes of 300 µL of 50% acetonitrile, followed by 2 washes of 300 µL of 100% acetonitrile. Each wash was carried out for 10 min at 25 °C with shaking at 1400 rpm. The gel slices were dried in a SpeedVac at 35 °C for 10 min. For trypsin digestion, the gel slices were pre-incubated with 7 µL of 15 ng/mL trypsin (Promega, Charbonnières-les-bains, France) at room temperature for 10 min. Afterwards, 25 µL of 50 mM ammonium bicarbonate was added, and the gel slices were incubated at 37 °C for 16 h. The peptide-containing supernatants were dried in a SpeedVac at 56 °C for 30 min, then resuspended in 20 µL of solution containing 0.05% formic acid and 3% acetonitrile for mass spectrometry experiments. The resulting peptides were analyzed with a nano-HPLC (serie 1200, Agilent Technologies, Montpellier, France) directly coupled to an ion-trap mass spectrometer (Bruker, Coventry, UK) equipped with a nano-electrospray source. The peptides were separated for 30 min on a gradient of 5 to 90% acetonitrile. The fragmentation voltage was 1.3 V. The resulting MS/MS spectra were searched against a human protein database using the Spectrum Mill Rev. A 03 03 84 SR4 software package (Agilent). To quantify a particular peptide in the sample, the ion trap was set in MRM (Multiple Reaction Monitoring) mode.

### 3.8. IPA Analysis

The tEBNA-LP partners were functionally annotated using IPA software (Ingenuity Systems, www.ingenuity.com). The dataset containing tEBNA-LP partners was uploaded into the application and each partner was mapped to its corresponding cellular function and disease category in Ingenuity Knowledge Base. The top functions and diseases were classified according to the number of molecules involved in these processes.

## 4. Conclusions

The main function of the full-length EBNA-LP is to activate the transcriptional activity of EBNA-2 in order to induce the expression of some cellular (Cyclin D2) [60] or viral (LMP-1) [19] genes. Our finding that the tEBNA-LP forms complexes with two cellular transcription factors (SSRP1 and TFEC) suggests that this truncated form of EBNA-LP, in addition to directly modulating the apoptotic pathway by interacting with effector proteins such as PP2A, could also regulate the expression of some cellular genes independently of EBNA-2.

## Figures and Tables

**Table 1:** tEBNA-LP partners identified by mass spectrometry.

**Cellular Proteins**	**Symbols**	**ID**	**Distinct Peptides**	**MS/MS Best Search Score (Identification Search)**
Transcription factor EC	TFEC	O14948	1	12.50
FACT complex subunit SSRP1	SSRP1	Q08945	6	89.72
Serine/threonine-protein phosphatase 2A	PP2A A	P30153	3	*
Tyrosine-protein phosphatase non-receptor type 13	PTPN13/FAP1	Q12923	2	23.39
Calcium-calmodulin independent nitric oxide synthase	iNOS	P35228	1	9.26
Cytoplasmic linker associated protein 1	CLASP1	Q7Z460	4	25.50
Fibronectin	FN1	P02751	14	165.44
Profilin-1	PFN1	P07737	10	137.87
α-2-macroglobulin	A2M	P01023	1	9.12
L-lactate dehydrogenase A chain	LDHA	P00338	19	301.06
Ceruloplasmin precursor	CP	P00450	2	33.22
Oxysterol-binding protein-related protein 3	OSBPL3	Q9H4L5	1	11.06
RING finger protein unkempt homolog	UNK	Q9C0B0	2	15.79
H/ACA ribonucleoprotein complex subunit 4	DKC1	O60832	1	7.55
Haptoglobin precursor	HP	P00738	1	13.57
10 kDa heat shock protein, mitochondrial	HSPE1	P61604	6	96.96
Fibrinogen gamma chain precursor	FGG	P02679	4	53.50
Fibrinogen beta chain precursor	FGB	P02675	10	15.81
Putative heat shock protein HSP 90-β-3	HSP90	Q58FF7	19	202.04
Ezrin	EZR	P15311	24	199.31
L-lactate dehydrogenase B chain	LDHB	P07195	18	285.01
Exosome complex exonuclease RRP44	DIS3	Q9Y2L1	14	160.93
Glycogen debranching enzyme	AGL	P35573	9	112.33
Pre-mRNA branch site protein p14	SF3B14	Q9Y3B4	2	41.10
Tight junction protein ZO-2	TJP2	Q9UDY2	3	14.13
Histone-lysine N-methyltransferase	MLL	Q03164	2	17.19
GTPase-activating Rap/Ran-GAP domain-like protein 3	GARNL3	Q5VVW2	1	14.75
Laminin subunit α-4	LAMA4	Q16363	1	12.71
**Viral Proteins**	**Symbol**	**ID**	**Distinct Peptides**	**Best Score**
Epstein-Barr nuclear antigen leader protein	EBNA-LP	Q1HVI8	2	42.68
Portal protein	BBRF1	Q1HVF2	2	7.74
Ribonucleoside-diphosphate reductase large subunit	BORF2	P0C702	4	13.19
Immediate-early phosphoprotein Serine/threonine-protein kinase	BGLF4	P0C6Z8	1	7.68
Epstein-Barr nuclear antigen 4	EBNA-3B	Q1HVG4	1	49.95

* PP2A was identified twice in MRM search but never in identification search, accordingly no score for PP2A is presented. Figure S1 (Supplementary Materials) shows the MS/MS spectra of the PP2A peptides detected by MRM.

**Table 2:** Molecular and cellular functions of tEBNA-LP partners.

Cellular Functions	Molecules	Numbers of Molecules
Cell Death	EZR, FN1, iNOS, PP2A A, PTPN13/FAP1, LAMA4, HSPE1, SF3B14, A2M, SSRP1, LDHA, MLL.	12
Cellular Growth and Proliferation	FN1, LDHA, iNOS, PP2A A, PTPN13/FAP1, PFN1, TJP2, LAMA4, EZR, DKC1, A2M, MLL.	12
Cellular Development	EZR, FN1, PFN1, PTPN13/FAP1, PP2A A, LAMA4, iNOS, A2M, LDHA, MLL.	10
Cell Signaling	FGB, FGG, FN1, iNOS, HP, PP2A A, PFN1, A2M, MLL.	9
Cellular Movement	EZR, FN1, iNOS, HP, PFN1, FGB, SSRP1, A2M.	8
Cell-To-Cell Signaling and Interaction	FGG, FN1, TJP2, CP, iNOS, A2M.	6
Gene Expression	SSRP1, TFEC, FN1, iNOS, A2M.	5
Post-Translational Modification	HSPE1, PP2A A, PTPN13/FAP1, CP, MLL.	5
Cell Morphology	LAMA4, FN1, PFN1, EZR, A2M.	5
Amino Acid Metabolism	CP, PP2A A, PTPN13/FAP1, iNOS.	4

## Supplementary Materials

The following are available online at http://www.mdpi.com/2072-6694/10/1/12/s1. Figure S1: MS/MS spectra of the PP2A peptides SALASVIMGLSPILGK and QLSQSLLPAIVELAEDAK detected by MRM in the immunoprecipitates, Table S1: Categories of disease involving the tEBNA-LP partners.
