# Supplementary material for: New Interactors of the Truncated EBNA-LP Protein Identified by Mass Spectrometry in P3HR1 Burkitt’s Lymphoma Cells
Source: Cancers (Basel). 2018 Jan 5;10(1):12. doi: 10.3390/cancers10010012 (PMC5789362; doi:10.3390/cancers10010012)
Supplement: Supplementary file 1 [file cancers-10-00012-s001.pdf]

cancers

MDPI

Supplementary Materials: New Interactors of the Truncated EBNA-LP Protein Identified by Mass Spectrometry in P3HR1 Burkitt's Lymphoma Cells

Sonia Chelouah, Emilie Cochet, Sophie Couvé, Sandy Balkaran, Aude Robert, Evelyne May, Vasily Ogryzko and Joëlle Wiels

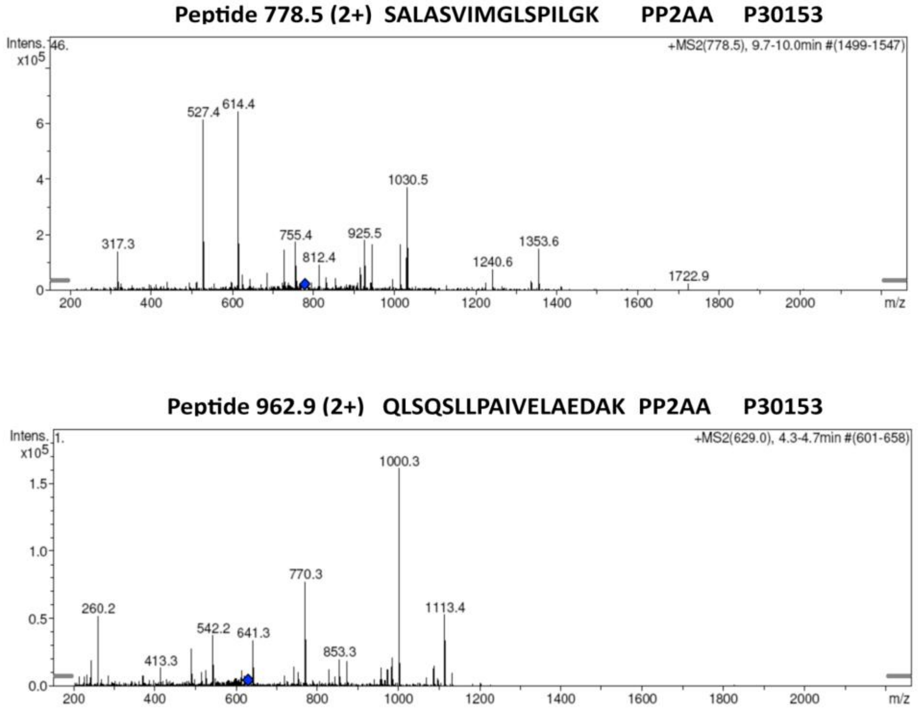

Figure S1. MS/MS spectra of the PP2A peptides SALASVIMGLSPILGK and QLSQSLLPAIVELAEDAK detected by MRM in the immunoprecipitates.

Table S1. Categories of disease involving the tEBNA-LP partners

Categories of disease	Molecules	Numbers of molecules
Genetic Disorder	FGB, FGG, CP, HP, LDHA, LDHB, PFN1, SETDB1, DKC1, AGL, FN1, GARNL3, HSPE1, LAMA4, LOC57653, NOS2, OSBPL3, PTPN13/FAP1, TJP2, MLL, A2M.	21
Cancer	CP, EZR, FGG, FN1, HP, HSPE1, LAMA4, LDHA, NOS2, PFN1, DKC1, MLL, A2M, DIS3.	14
Neurological Disease	CP, FGG, GARNL3, HP, LDHA, LDHB, NOS2, PFN1, PTPN13/FAP1, SETDB1, TJP2, FN1, LAMA4, A2M.	14
Skeletal and Muscular Disorders	CP, FGG, HP, LDHA, LDHB, PFN1, SETDB1, FN1, NOS2, FGB, PPP2R1A, A2M.	11
Hematological Disease	FGB, FGG, LAMA4, FN1, PPP2R1A, NOS2, OSBPL3, DIS3, MLL.	9

© 2018 by the authors; licensee MDPI, Basel, Switzerland. This article is an open access article distributed under the terms and conditions of the Creative Commons by Attribution (CC-BY) license (http://creativecommons.org/licenses/by/4.0/)
